# Associations between urinary kidney injury biomarkers and cardiovascular mortality risk in elderly men with diabetes

**DOI:** 10.1080/03009734.2016.1192704

**Published:** 2016-06-17

**Authors:** Aleksandra Tonkonogi, Axel C. Carlsson, Johanna Helmersson-Karlqvist, Anders Larsson, Johan Ärnlöv

**Affiliations:** a Department of Medical Sciences, Cardiovascular Epidemiology, Uppsala University, Uppsala, Sweden;; b Division of Family Medicine, Department of Neurobiology, Care Sciences and Society, Karolinska Institutet, Huddinge, Sweden;; c Department of Medical Sciences, Biochemical Structure and Function, Uppsala University, Uppsala, Sweden;; d School of Health and Social Studies, Dalarna University, Falun, Sweden

**Keywords:** Biomarkers, diabetes, epidemiology, kidney, KIM-1

## Abstract

**Aim:**

Three urinary biomarkers, kidney injury molecule-1 (KIM-1), neutrophil gelatinase-associated lipocalin (NGAL), and cystatin C, have been suggested as clinically relevant highly specific biomarkers of acute kidney tubular damage. Yet, the utility of these biomarkers in the prognostication of diabetic nephropathy has been less studied. Therefore, we aimed to investigate the longitudinal association between these urinary biomarkers and cardiovascular mortality in patients with diabetes.

**Methods:**

The study sample consisted of participants with diabetes in the community-based Uppsala Longitudinal Study of Adult Men (*n* = 91; mean age 77.8 years). During follow-up (median 8.3 years, interval 0.7–13.4 years), 33 participants died of cardiovascular causes.

**Results:**

In a multivariable Cox regression model adjusting for age, glomerular filtration rate, and urinary albumin/creatinine ratio, higher urinary KIM-1/creatinine was associated with an increased risk for cardiovascular mortality (HR per SD increase 1.51, 95% confidence intervals 1.03–2.24, *P* = 0.03). Neither urinary NGAL/creatinine nor urinary cystatin C/creatinine were independently associated with an increased cardiovascular mortality risk.

**Conclusion:**

In elderly men with diabetes, higher urinary KIM-1/creatinine was associated with an increased long-term risk of cardiovascular mortality independently of established markers of diabetic nephropathy. Our data provide support for kidney tubular damage as an important aspect of diabetic nephropathy that merits further investigation.

## Introduction

The prevalence of diabetes mellitus and diabetic nephropathy is escalating globally (1). The underlying pathology leading to diabetic nephropathy is multifactorial, and tubular damage appears early in disease development and may precede glomerular damage (2). Yet, to date, the role of kidney tubular damage in diabetic nephropathy is incompletely understood. The currently used established kidney disease biomarkers for diagnosing diabetic nephropathy, urinary albumin/creatinine ratio and creatinine-based estimated glomerular filtration rate, have limited sensitivity and specificity as they are influenced by many extra-renal factors.

Therefore, further exploration of specific tubular damage biomarkers in diabetic nephropathy may prove to be clinically important. Urinary kidney injury molecule-1 (uKIM-1) (3,4), neutrophil gelatinase-associated lipocalin (uNGAL) (5,6), and cystatin C (uCystC) (7,8) have all been suggested as highly specific biomarkers for acute tubular damage; however, the aforementioned biomarkers may also indicate chronic tubular damage. Yet, data on long-term prognostic importance of these biomarkers in patients with diabetes are limited.

KIM-1 is a transmembrane protein, almost exclusively expressed on the apical surface of proximal tubule cells as a response to injury, and its expression does not disappear until the damage is totally resolved (3). NGAL is a protein which is a part of the lipocalin superfamily (5), expressed in the thick ascending limb of Henle’s loop and collecting ducts as a response to injury.

Cystatin C is freely filtered through glomeruli (9) and thereafter reabsorbed and degraded in the proximal tubuli. Thus, only trace amounts can be found in the urine from healthy kidneys (10).

In the present study, we hypothesized that chronic kidney tubular damage may increase the risk of macrovascular complications in patients with diabetes. Accordingly, we aimed to investigate the association between three kidney tubular damage biomarkers and the risk for cardiovascular mortality in a study population of elderly men with diabetes.

## Material and methods

The Uppsala Longitudinal Study of Adult Men (ULSAM) was used as study samples. The design has been described previously (http://ww.pubcare.uu.se/ULSAM) (11–13). The analyses were based on the fourth examination cycle in 1997–2001, when the men were approximately 77 years old (*n* = 838). Complete data for the subgroup of individuals with diabetes were available for 91 participants. A written informed consent was given by all participants, and the study protocol was approved by the ethics committee of Uppsala University.

### Medical information

Participants with diabetes at baseline were defined using the following criteria: use of diabetes medication including oral medication or insulin, or a fasting glucose ≥7.0 mmol/L. Information about smoking habits and ongoing medications was obtained using questionnaires. Height (cm), body (kg), waist circumference (cm), BMI (kg/m^2^), hip circumference (cm), and waist–hip ratio were measured. Blood pressure (mmHg) was determined in the right arm with the subject in supine position after 10 min of rest.

### Clinical and biochemical analyses

All blood samples, venous, were drawn in the morning after an overnight fast (14). Twenty-four-hour urinary samples were collected. Urinary KIM-1 was analyzed (the cleaved extracellular part which can be found in the urine) using a commercial ELISA kit (DY1750 R&D Systems, Minneapolis, MN, USA). Urinary NGAL was analyzed using an ELISA kit (DY1757, R&D Systems, Minneapolis, MN, USA). Urine albumin was measured by nephelometry (urine albumin, Dade Behring, Deerfield IL, USA), using a Behring BN ProSpec^®^ analyzer (Dade Behring). Creatinine in the urine was measured by means of a modified kinetic Jaffe reaction on an Architect Ci8200^®^ analyzer (Abbott, Abbott Park, IL, USA), and the aforementioned values were used to calculate urinary albumin–creatinine ratio (uACR). The analysis of urinary cystatin C was carried out using a Mindray BS-380 (Shenzhen Mindray Bio-medical Electronics, Shenzhen, China) with reagents from Gentian (Moss, Norway). Serum cystatin C was analyzed with a latex-enhanced reagent (N Latex Cystatin C, Dade Behring, Deerfield, IL, USA) with a Behring BN ProSpec analyzer (Dade Behring). HbA_1c_(%) was analyzed using a high-pressure liquid chromatograph with a gradient system (BIO-RAD Laboratories). HDL-cholesterol (mmol/L) was measured by separating the particles by precipitation with chloride and phosphotungstate. Total cholesterol (mmol/L) in serum was analyzed using enzymatic techniques, the IL Test Cholesterol Trinder’s Method 181618-10 in a Monarch apparatus (Instrumentation Laboratories, Lexington, USA). Plasma glucose was measured using the glucose dehydrogenase method (Gluc-DH, Merck, Darmstadt, Germany). Estimated glomerular filtration rate (eGFR) was calculated using serum cystatin C and by applying the formula *y* = 77.24*x* – 1.2623. For one of the Cox proportional hazard models albuminuria was dichotomized at 3 g/mmol creatinine.

All urinary biomarkers have been presented as a ratio to urinary creatinine to compensate for varying urine concentrations between the patients.

### Outcome

Information about death due to cardiovascular disease was obtained from the Swedish cause-of-death register and defined as International Classification of Diseases (10th Revision) codes I00–I99.

### Statistical analysis

To investigate associations between cardiovascular mortality and the novel kidney injury biomarkers multivariable Cox proportional hazard models were used. Model A: crude; Model B: adjusted for uACR; Model C: adjusted for eGFR; and Model D: adjusted for uACR and eGFR. Additional Cox proportional hazard analyses were carried out as secondary analyses, one including uKIM-1/Cr and known cardiovascular risk factors (systolic blood pressure, hypertension treatment, total cholesterol, HDL, lipid-lowering medication, smoking, previous cardiovascular event, HbA_1c_, diabetes medication, uACR, eGFR, and atrial fibrillation). Moreover, Cox proportional hazard models with all the three kidney injury biomarkers and uACR and eGFR and a model with KIM-1/Cr as a dichotomous variable, below or above 175 ng/mmol, microalbuminuria, and chronic kidney disease CKD were carried out.

## Results

Baseline characteristics can be seen in Table 1, which gives us information about levels of the urinary biomarkers, smoking habits, relevant medication, and much more.

**Table 1.** Baseline characteristics.

Variable	Mean	SD
A: Continuous variables		
uKIM/Cr (ng/mmol)	121.4	10.2
uNGAL/Cr (ng/mmol)	2050.4	261.4
uCyst/Cr (ng/mmol)	14.0	0.003
Age (years)	77.8	0.17
Systolic blood pressure (mmHg)	152	4.43
Total cholesterol (mmol/L)	5.15	0.14
HDL(mmol/L)	1.16	0.04
BMI (kg/m^2^)	27.3	0.53
HbA_1c_ (Mono S, %)	5.8	0.12
GFR-cyst (mL/min/1.73 m^2^)	73.5	2.5
uACR (mg/mmol)	1.5	0.33
	*n*	%
B: Categorical variables		
Smoking	4	4
Hypertension treatment	61	67
Lipid-lowering treatment	23	25
Diabetes treatment	47	52
Previous CVD	7	8
Atrial fibrillation	12	14
Microalbuminuria	33	36
Chronic kidney disease	20	22

BMI: body mass index; GFR: glomerular filtration rate; HDL: high-density lipoprotein; previous CVD: previous cardiovascular disease events; uACR: urinary albumin–creatinine ratio; uCystC/Cr: urinary cystatin C–creatinine ratio; uKIM-1/Cr: urinary kidney injury molecule-1–creatinine ratio; uNGAL/Cr: urinary neutrophil gelatinase-associated lipocalin–creatinine ratio.

During follow-up (median 8.3 years, interval 0.7–13.4 years) 33 participants died of cardiovascular causes (incidence rate of 4.7 per 100 person years at risk). In Cox proportional hazard analyses, adjusting for age and established kidney disease biomarkers (eGFR and uACR), it was found that higher uKIM-1/Cr was significantly associated with an increased risk for cardiovascular mortality (Table 2).

**Table 2.** The association between urinary biomarkers and the risk for cardiovascular mortality: multivariate Cox proportional hazard regression.

Variable	Model A Hazard ratio (95% CI)	Model B Hazard ratio (95% CI)	Model C Hazard ratio (95% CI)	Model D Hazard ratio (95% CI)
uKIM-1/Cr	1.58 (1.13–2.22)	1.51 (1.04–2.19)	1.50 (1.08–2.10)	1.52 (1.03–2.24)
	***P* = 0.008**	***P* = 0.032**	***P* = 0.016**	***P* = 0.034**
uNGAL/Cr	1.07 (0.78–1.48)	0.99 (0.71–1.39)	1.07 (0.76–1.48)	0.98 (0.68–1.43)
	*P* = 0.66	*P* = 0.95	*P* = 0.71	*P* = 0.93
uCystC/Cr	1.26 (1.00–1.60)	1.16 (0.86–1.56)	1.09 (0.85–1.40)	1.02 (0.75–1.39)
	***P* = 0.049**	***P* = 0.033**	*P* = 0.50	*P* = 0.89

*P* values: significant values in bold.

Model A: no adjustments; Model B: adjusted for uACR; Model C: adjusted for eGFR; Model D: adjusted for uACR and eGFR.

In secondary analyses, uKIM-1/Cr was still associated with cardiovascular mortality when established cardiovascular risk factors (systolic blood pressure, hypertension treatment, total cholesterol, HDL cholesterol, lipid-lowering medication, smoking, previous cardiovascular disease, HbA1c, diabetes medication) were added to Model D with adjustments for both uACR and eGFR (HR per standard deviation (SD) increase of uKIM-1/Cr 1.82, 95% CI 1.13–2.95, *P* = 0.01). Higher uCystC/Cr was associated with an increased risk for CVD mortality in age-adjusted models, but this association was attenuated and no longer statistically significant after adjustment for both uACR and eGFR (Table 2). Urinary NGAL/Cr was not associated with increased CVD mortality in any model (Table 2). In a model including all three urinary biomarkers as well as eGFR and uACR, only uKIM-1/Cr and eGFR remained associated to cardiovascular mortality (HR per SD increase of eGFR 0.58, 95% CI 0.37–0.89, *P* = 0.01; and for uKIM-1/Cr 1.54, 95% CI 1.03–2.30, *P* = 0.03).

## Discussion

In the present study, higher ratios of uKIM-1/Cr were associated with an increased risk of cardiovascular mortality even after adjusting for established kidney disease markers and cardiovascular risk factors. Neither urinary NGAL/Cr nor urinary Cystatin C/Cr were independently associated with CVD mortality risk in multivariable models. Our data provide additional support for kidney tubular damage as an important aspect of diabetic nephropathy that merits further investigation.

There are a few previous community-based studies that have reported an association between tubular damage biomarkers and cardiovascular mortality (15–18). However, previous studies investigating the association between the urinary kidney biomarkers and mortality risk in patients with diabetes are scarce and inconsistent. In one study, KIM-1/Cr did not provide additional prognostic value for 4-year mortality risk after being adjusted for established CVD risk factors in 978 patients with type 2 diabetes (16). In contrast, a recent study in Pima Indians with type 2 diabetes showed that higher urinary KIM-1 was associated with increased long-term risk of mortality but not end-stage renal disease (19). We are not aware of any previous studies reporting the association between urinary KIM-1 and cardiovascular mortality in patients with diabetes. Perhaps differences between previous studies and our study may be due to differences in outcome (total versus CVD mortality), length of follow-up, sampling procedure (spot sample versus 24-hour collection of urine) or differences in age, ethnicity, and gender distribution of the study samples.

Several mechanisms may explain the development of tubular damage in diabetes. First, tubular hypertrophy has been observed already after days of increased glucose levels (2). Tubular cells are particularly vulnerable since their glucose uptake is insulin-independent (2). Furthermore, hypertrophied cells are maintained in the G1 phase of the cell cycle (20) and are consequently more vulnerable to apoptotic stimuli. Hyperglycemia also activates many growth factors. For example, TGFβ, which can be stimulated by advanced glycation end-products and angiotensin II (21,22), has been shown to be involved in the development of tubular hypertrophy (21). It has also been shown that higher urinary albumin may directly damage the tubuli through induction of pro-inflammatory and pro-fibrotic changes (23), as well as apoptosis of the tubular cells. Tubular hypoxia has also been shown to be associated with nephropathy, and has been proposed to be a fundamental factor underpinning development of chronic kidney disease and end-stage renal disease (24). Yet, we cannot say if hypoxia is mirrored by the KIM-1 levels in the individuals with diabetes in the present study.

The causal link between chronic tubular damage and the development of cardiovascular disease is less clear. When kidney function declines, calcium, potassium, urea, and various harmful proteins accumulate; however, this may be more relevant in later stages of kidney disease. Accumulation of harmful substances may in turn lead to accelerated atherosclerosis and hence CVD. Furthermore, tubular damage may result in an increased reabsorption of sodium and water in the proximal tubuli (2), which may lead to hypertension.

Neither urinary NGAL nor urinary cystatin C could be linked to an increased risk of cardiovascular mortality. This may partially be explained by the fact that NGAL expression is not only limited to the kidneys as is the case with KIM-1. NGAL is also a biomarker for inflammation. It is noteworthy that cystatin C is taken up by the proximal tubule cells and then degraded (7,8). Thus, findings of cystatin C in the urine may mirror tubular dysfunction rather than specific tissue damage.

The increased levels of urinary KIM-1, as seen in patients with diabetes, may also be the result of reverse causation, meaning that CVD itself results in higher levels of uKIM-1. It has been shown that patients with prevalent congestive heart failure have higher levels of KIM-1 compared to healthy controls (25).

Diabetic nephropathy is a common and detrimental complication of diabetes leading to increased morbidity and mortality, mainly due to CVD. Therefore, it is of great clinical importance to evaluate novel kidney injury biomarkers, which may signal kidney damage at an earlier stage than microalbuminuria and reduced GFR. Interestingly, previous studies have reported that elevated levels of KIM-1/Cr correlate with the progression from normoalbuminuria to microalbuminuria (14) and that urinary KIM-1/Cr is associated with insulin resistance even prior to the development of diabetes (26), indicating that urinary KIM-1/Cr could be an early marker for diabetic nephropathy.

It is possible that KIM-1 could be used to monitor the progression or regression of nephropathy since levels of KIM-1 in the urine decrease as a response to RAAS blockade (27). However, it is not fully known if reduced levels of KIM-1 will actually lead to decreased cardiovascular risk (15). Importantly, the clinical relevance of KIM-1 screening in patients with diabetes remains to be established, and further large-scale studies are needed.

The main strength of our study is the longitudinal study design with up to 13 years of follow-up. The main limitation is the small study sample with few events during follow-up, which led to limited statistical power to detect modest associations between kidney biomarkers and outcome. Yet, regardless of the limited statistical power, uKIM-1/Cr predicted cardiovascular mortality in all models. As the study participants were elderly men, extrapolations of our findings to women and other age groups and ethnicities have to be done cautiously. We did not distinguish between type of diabetes; however, diabetes type 2 is dominating among 77-year-old men in the community, and we suggest that the results should be interpreted as if all individuals had type 2 diabetes.

In conclusion our data put forward urinary KIM-1/creatinine as a promising biomarker for diabetic nephropathy and provide additional support for the importance of early tubular damage as part of the underlying pathology of diabetic nephropathy.
